# Aggressive papillary adenocarcinoma on atypical localization: A unique case report

**DOI:** 10.1097/MD.0000000000004110

**Published:** 2016-07-18

**Authors:** Mecdi Gurhan Balci, Mahir Tayfur, Ayse Nur Deger, Orhan Cimen, Huseyin Eken

**Affiliations:** a Department of Pathology, Mengucek Gazi Education and Research Hospital, Erzincan University; b Department of Pathology, Evliya Celebi Education and Research Hospital, Dumlupinar University, Kutahya; c Department of General Surgery, Mengucek Gazi Education and Research Hospital, Erzincan University, Turkey.

**Keywords:** aggressive digital papillary adenocarcinoma, atypical localization, scalp

## Abstract

**Introduction:**

Aggressive digital papillary adenocarcinoma (ADPA) is a rare sweat gland tumor that is found on the fingers, toes, and the digits. To date, <100 cases have been reported in the literature. Apart from 1 case reported in the thigh, all of them were on digital or nondigital acral skin.

**Case presentation:**

A 67-year-old Caucasian woman was admitted to the hospital due to a mass on the scalp. This lesion was present for almost a year. It was a semimobile cyctic mass that elevated the scalp. There was no change in the skin color. Its dimensions were 1.5 × 1 × 0.6 cm. The laboratory, clinic, and radiologic findings (head x-ray) of the patient were normal. It was evaluated as a benign lesion such as lipoma or epidermal cyst by a surgeon due to a small semimobile mass and no erosion of the skull. It was excised by a local surgery excision. The result of the pathologic examination was aggressive papillary adenocarcinoma. This diagnosis is synonymous with ADPA.

**Conclusion:**

In our case, localization was scalp. This localization is the first for this tumor in the literature. In addition, another atypical localization of this tumor (ADPA) is thigh in the literature. This case was presented due to both the rare and atypical localizations. That is why, in our opinion, revision of “digital” term in ADPA is necessary due to seem in atypical localizations like thigh and scalp.

## 1 Introduction

Sweat gland carcinomas are rare malignant tumors of the skin. The well-defined entities porocarcinoma, microcystic adnexal carcinoma, aggressive digital papillary adenocarcinoma (ADPA), mucinous eccrine carcinoma, adenoid cystic carcinoma, spiradenocarcinoma, cylindrocarcinoma, and hidradenocarcinoma are described.^[1,2]^

In 1979 Helwig from the Armed Force Institute of Pathology introduced a term called aggressive digital papillary adenoma and presented 22 cases under this term at the American Academy of Clinical and Pathologic Conference in Chicago^[3]^ ADPA first described by Helwig in 1984 and later by Kao GF in 1987.^[4]^

ADPAs are characterized by lesions on the fingers, toes, and the digits. These tumors are capable of aggressive local invasion resulting in a high recurrence rate. As the tumor is slow-growing, painless, and clinically inconspicuous, the diagnosis is often missed or delayed.^[4–7]^

The lesion is serious but often overlooked because it is confused clinically with benign and nontumorous entities.^[5]^

ADPA is characterized by aggressive biological behavior, with a relatively high potential for local recurrence (30–40% of cases) and distant metastasis (up to 14%). Complete surgical excision is the treatment of choice for ADPA; however, there are no uniform diagnostic guidelines or recognized effective treatments for metastasis, and no therapeutic targets have been identified.^[8]^

Despite documented spread to regional lymph nodes, the information on sentinel lymph node status in ADPA is reported infrequently, with only 1 documented case of positive findings.^[9]^

Less than 100 cases have been reported. The majority of these cases are described in males in their fifth to seventh decade.^[10]^ The median age of occurrence is 52 years. It is rare in females with male:female ratio of 9:1.^[5,11]^

Histologically ADPA has solid and cystic spaces, with or without papillary projections in the cystic spaces. The cysts are formed either by central degeneration/necrosis of solid areas or amassed secretory material, which appeared to be mucin.^[12]^ Tumor features include a grenz zone, fibrocollagenous stroma, a mixed tubuloalveolar and papillary pattern, and focal squamous metaplasia.^[13]^

The differential diagnosis comprises apocrine adenocarcinoma, adenoid cystic carcinoma of the sweat glands, and mucinous eccrine carcinoma.^[4]^

### 1.1 Case presentation

A 67-year-old Caucasian woman admitted to hospital due to a mass on the scalp. This lesion was present for almost a year. It was a semimobile cyctic mass that elevated the scalp. There was no change in the skin color. Its dimensions were 1.5 × 1 × 0.6 cm. The laboratory, clinic, and radiologic findings (head x ray) of the patient were normal. It was evaluated a benign lesion such as lipoma or epidermal cyst by surgeon due to a small semimobile mass and no erosion of the skull. It was excised by a local surgery excision. The skin is sent as a different part from this excision material.

**Figure 1:**
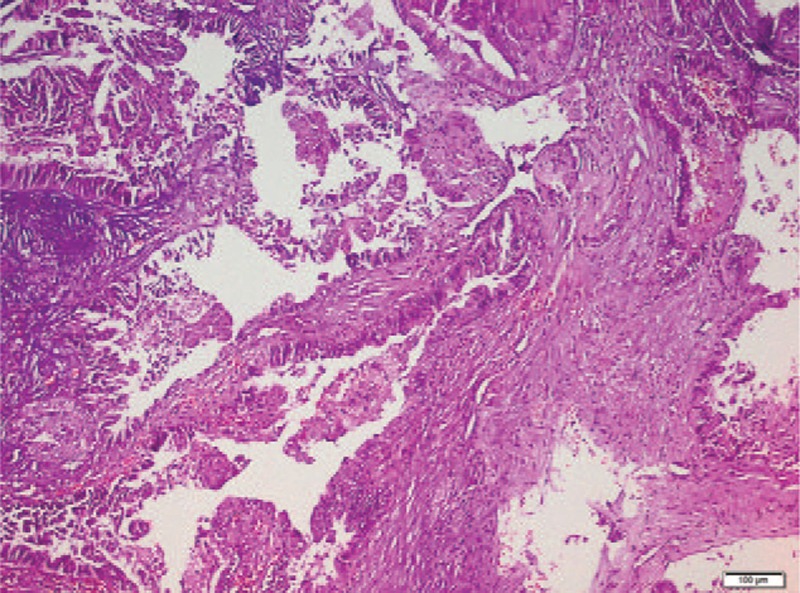
Solid and cystic spaces including tubuloalveolar, ductal, and papillary structures protruding into cistically dilated lumina (HE× 100).

**Figure 2:**
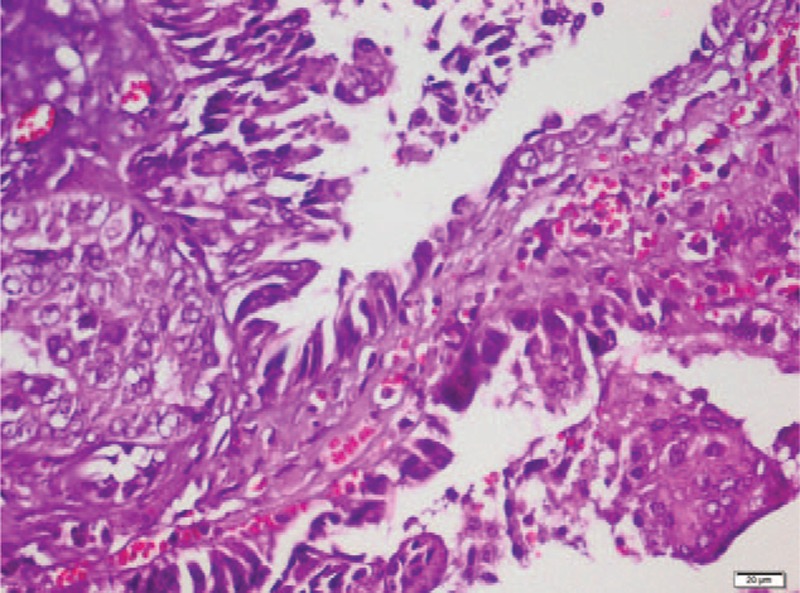
Atypical epithelial cells including hyperchromatic, pleomorfic, and big nucleus (HE× 400).

**Figure 3:**
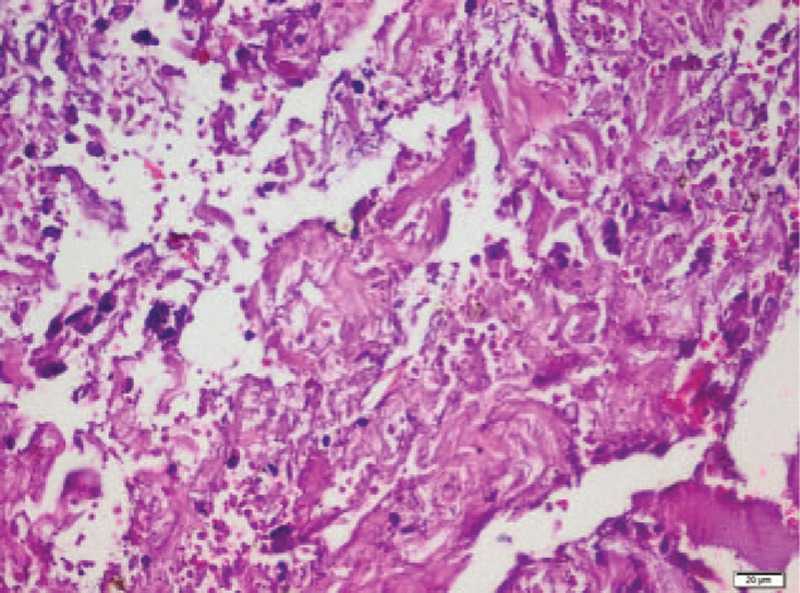
Necrotic areas (HE× 400).

In pathological examination, there were solid and cystic spaces including tubuloalveolar, ductal, and papillary structures protruding into cistically dilated lumina (Fig. 1). These structures were lined by atypical epithelial cells including hyperchromatic, pleomorfic, and big nucleus (Fig. 2). Necrotic areas and mitotic figures were present (Fig. 3). There was no epithelial invasion.

These histopathologic findings were compatible with ADPA. For elimination of a metastatic primary papillary carcinoma focus such as lung, breast, thyroid, etc. An immunohistochemical study was performed. EMA, PanCK, and CEA were positive. SMA, Calponin, and P63 were negative. These immunohistochemical dyes support diagnosis of ADPA. In the examination for elimination of probably a metastatic focus, TTF1 for lung, ER and PR for breast, CK19 and HBME1 for thyroid, and GFAP for brain were negative. In clinical investigation before pathologic report for the ADPA, it is approved that there was not any mass in lung, breast, thyroid, brain, and ovary. The patient is in the 4th month in the postoperative period and there was no pathologic finding.

## 2 Discussion

Is ADPA really aggressive? Does it only occur at digital location? Does it always have microscopic papillary features? Is it really adenocarcinoma? This kind of lesion must have in summary, the so-called ADPA is not an aggressive tumor. Although most if not all cases reported on digital or nondigital acral skin in the literature were from, there is no reason to believe that this kind of lesion only occurs on digits or is restricted to acral skin.^[14]^ Almost 100 cases have been reported in the literature. Apart from 1 case reported in the thigh, all of them were on digital or nondigital acral skin.^[15]^

In our case, localization was scalp. This localization is the first for this tumor in the literature. In addition, another atypical localization of this tumor (ADPA) is thigh as reported in the literature.^[14,15]^

According to Sheng Chen and Masoud Asgari, it does not always have microscopic papillary feature and furthermore it might be adenomyoepithelial tumor rather than adenocarcinoma. Thus, the so-called aggressive digital papillary adenocarcinoma is not really ADPA.^[14]^

Even according to Duke et al^[16]^, the typical ADPA is “multinodular, solid, and cystic with papillary projections present in the cystic spaces. Twelve (18%) of the neoplasms were essentially only solid, lacking cystic spaces. Characteristic of all lesions was a pattern of fused back-to-back glands lined by cuboidal to low columnar epithelial cells in the solid portion of the tumors.”

In our case SMA and Calponin were negative. Although this condition supports malignity, it is not exact. Because the presence of SMA-positive and Calponin-positive myoepithelial cells around glandular structures has been thought of as a feature of benignity in the context of cutaneous adnexal tumors. In some primary cutaneous mucinous carcinoma, adenoid cystic carcinoma, and adenomyoepithelial carcinoma (also called malignant adenomyoepithelioma), myoepithelial layer might be found. In primary cutaneous mucinous carcinoma, histologically, an in situ component in the form of epithelial islands bounded by a myoepithelial layer, and highlighted by CK 5/6, p53, calponin and SMA, is frequently present in PCMC in comparison to metastatic mucinous carcinoma.^[17]^ In adenoid cystic carcinoma, inconstant expression of actin was described previously, which is possibly an indication of the presence of a myoepithelial cell population.^[18]^ In adenomyoepithelial carcinoma (also called malignant adenomyoepithelioma), immunohistochemical staining was done and clearly showed the presence of both epithelial and myoepithelial cells.^[19]^

The presence of tumor-associated myoepithelial cells should not be construed as an indication of benignity but rather another indication for a primary adnexal tumor should metastasis be a clinical or diagnostic consideration. The presence of tumor-associated myoepithelial cells histologically and immunohistochemically was not synonymous with benignity. As the name implies a malignant neoplasm, the rubric “aggressive” is unnecessary.^[19]^

The differential diagnosis of ADPA comprises apocrine adenocarcinoma, adenoid cystic carcinoma of the sweat glands, and mucinous eccrine carcinoma. Microscopic features were distinct from those of other eccrine sweat gland tumors and often led to the diagnosis of such metastatic carcinoma as that of the breast, lung, thyroid, and ovary.

The characteristic histologic features of ADPA included tubuloalveolar and ductal structures with areas of papillary projections protruding into cystic lumina. The stroma varied from thin, fibrous septae to areas of dense, hyalinized collagen.^[4]^

In our case, in the pathological examination, there were solid and cystic spaces including tubuloalveolar, ductal, and papillary structures protruding into cistically dilated lumina. These structures were lined by atypical epithelial cells including hyperchromatic, pleomorfic, and big nucleus. Necrotic areas and mitotic figures were present. These findings were typically aggressive digital papillary adenocarcinoma.

There were not apparent apocrine cells in our case; thus, apocrine adenocarcinoma was not though. In adenoid cystic carcinoma of the sweat glands and mucinous eccrine carcinoma, apparent papillary projections were not available. Thus, adenoid cystic carcinoma of the sweat glands and mucinous eccrine carcinoma were not though due to apparent papillary projections were avaliable in our case.

Metastatic papillary carcinoma to skin from breast, lung, thyroid, and ovary should be considered when the pathologic report is prepared.^[20]^ The immunohistochemical panel was supported to distinguish the aggressive digital papillary adenocarcinomas from metastatic malignities. In addition, it is approved that there was no mass in lung, breast, thyroid, brain, ovary, and elsewhere.

## 3 Conclusions

ADPA are rare sweat gland tumors. They were found on the fingers, toes, and the digits. Almost 100 cases have been reported in the literature. Apart from 1 case reported in the thigh, all of them were reported on digital or nondigital acral skin. In our case, localization was scalp. In the literature, this localization is the first for this tumor. This case was presented due to this condition. That is why, in our opinion, revision of “digitally” term in ADPA is necessary due to seem in atypical localizations such as thigh and scalp.

## Acknowledgment

The authors thank to the editor of *The American Journal of Dermatopathology* for suggested a convenient journal to us manuscript.
